# An Assessment of Enhancement Patterns in Abnormal Parathyroid Glands on Three-Phase CT Imaging

**DOI:** 10.7759/cureus.40166

**Published:** 2023-06-09

**Authors:** Joseph Vance-Daniel, Oliver Curwen, Lauren Stroud, Visvalingham Gnanananthan, Kashif Burney, Karim Jamal

**Affiliations:** 1 General Surgery, Epsom and St. Helier University Hospitals, National Health Service (NHS) Trust, London, GBR; 2 Radiology, Epsom and St. Helier University Hospitals, National Health Service (NHS) Trust, London, GBR; 3 General and Endocrine Surgery, Epsom and St. Helier University Hospitals, National Health Service (NHS) Trust, London, GBR

**Keywords:** head and neck and endocrine, four-dimensional computed tomography (4dct), ectopic parathyroid, parathyroid disease, atypical parathyroid

## Abstract

Background

Four-dimensional computed tomography (4DCT) is increasingly used in the investigation of primary hyperparathyroidism. The objective of this study was to identify and analyse the usefulness of different enhancement patterns on 4DCT to improve its sensitivity.

Methodology

Retrospective data were collected on 100 glands. A consultant head and neck radiologist measured the Hounsfield units (HU) of the parathyroid gland and surrounding normal thyroid tissue in the pre-contrast, arterial and venous phases. Each gland was grouped according to the enhancement pattern, and the percentage change in HU was also calculated between the three phases.

Results

Thirty-five parathyroid glands demonstrated enhancement higher than the thyroid gland in the arterial phase and lower in the delayed phase and were placed into group A. Four parathyroid glands demonstrated enhancement higher than the thyroid gland in the arterial phase and also higher in the delayed phase and were placed into group B. Fifty-nine parathyroid glands demonstrated enhancement lower than the thyroid gland in the arterial phase and also lower in the delayed phase and were placed into group C. Two parathyroid glands demonstrated enhancement lower than the thyroid gland in the arterial phase and higher in the delayed phase and were placed into group D.

Conclusions

This study demonstrated that the classically described enhancement pattern of the parathyroid gland is not always present or the most frequent, thereby showing that the enhancement pattern cannot be relied upon in isolation. Instead, a thorough understanding of anatomy, embryology and possible ectopic gland locations is essential.

## Introduction

Typically, there are four parathyroid glands: two superior and two inferior. However, the number can vary among individuals, with as few as one and up to 11 glands reported [1]. The inferior and superior parathyroid glands develop from the third and fourth pharyngeal pouches, respectively. The superior parathyroid glands are typically located on the posterior surface of the thyroid gland, within 1 cm of the junction of the recurrent laryngeal nerve (RLN) and inferior thyroid artery (ITA), at the level of the cricothyroid junction and positioned posteriorly to the RLN. Ectopic superior parathyroid glands may be found in the tracheoesophageal groove or para-oesophageal location and within the thyroid (intrathyroidal), the carotid sheath or posterior mediastinum [2]. The location of the inferior parathyroid glands is more variable due to the longer path of migration as they arise from the third branchial pouch but are commonly found inferior to the ITA and anteromedial to the RLN. Around 50% are found within 1 cm of the lower pole of the thyroid [3]. Ectopic glands may be found anywhere along the path of migration. Within the thymus (with which they migrate), perithymic fat, thyrothymic ligament or thyroid, anterior mediastinum and carotid sheath [4]. Patients with hyperparathyroidism are more likely to have ectopic parathyroid glands than those with normal parathyroid function [1]. This may be due to a phenomenon known as acquired ectopia - pathologically enlarged parathyroid glands may migrate due to the effects of gravity and regional dynamics on a gland as it increases in size and weight [3]. Due to the variation in the number and position of parathyroid glands, imaging plays a crucial role in the pre-operative workup of patients with hyperparathyroidism [5].

Four-dimensional computed tomography (4DCT) is becoming an increasingly used investigation in the management of primary hyperparathyroidism. First described in 2006 [6], 4DCT combines three-dimensional imaging with changes in contrast enhancement over time (the fourth dimension of the scan). The classically described enhancement pattern of the parathyroid gland is lower in attenuation than the thyroid gland in the non-contrast phase, higher attenuation than the thyroid (peak attenuation) in the arterial phase and lower attenuation than the thyroid in the delayed phase (washout phase) [7]. This enhancement pattern is heavily relied upon [8]; however, it may not always be present [9], resulting in some diagnostic uncertainty. Knowledge of alternative enhancement patterns could be beneficial in identifying parathyroid glands to allow for targeted operations. As this has not been investigated in depth previously, this study aimed to identify and analyse the usefulness of different enhancement patterns on 4DCT to improve the sensitivity of the 4DCT and reduce the diagnostic uncertainty, which may lead to a more targeted approach to parathyroid surgery.

## Materials and methods

Retrospective data were collected from patients who underwent pre-operative CT parathyroid scan and parathyroidectomy between August 2018 and May 2021 in a single trust, performed by a single consultant endocrine surgeon. Patients were identified using the British Association of Endocrine and Thyroid Surgeons (BAETS) audit, and a total of 92 patients were identified. Online hospital medical records were accessed to record age, sex, pathology reports, operation notes and imaging reports. An online picture archiving and communication system (PACS) was used to access images from CT scans. Patients were included if they had pre-operative triple-phase CT scans, had undergone a parathyroidectomy with histological results available and demonstrated pathologically proven adenoma or hyperplasia. Patients who did not undergo triple-phase CT scan pre-op and who had a scan performed in another trust where the protocol used was different were excluded. Other exclusion criteria included patients aged under 18 years or over 80 years and those who had been diagnosed with malignancy, undergoing chemotherapy or were deceased within six months of data collection.

**Figure 1:**
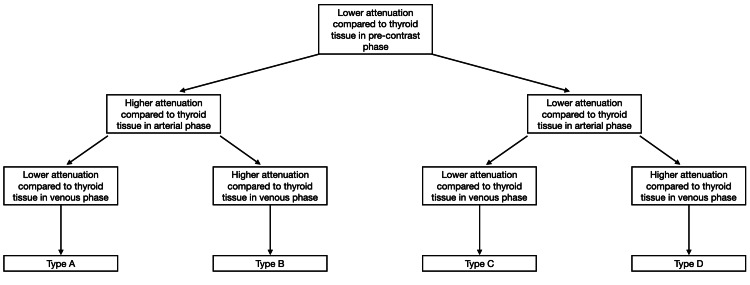
Allocation of subgroups by enhancement patterns. Figure credit: All authors.

All images were acquired using GE GSI Revolution / GE Evo HD 128 (GE Healthcare, Chicago, IL, USA) slice machines. Patients were in the supine position with a headrest with two scout films and centring on the sternal notch. Omnipaque 350 (80 mL) was mixed with 25 mL saline and injected at the rate of 4 mL/s through an 18- or 20-gauge Venflon (BD Switzerland Sarl, Eysins, Switzerland). Each patient was scanned from the angle of the mandible to the carina in three phases: pre-contrast at 0 seconds, arterial phase with a 25-second delay and venous phase with an 80-second delay. All the images were reviewed by a consultant radiologist, with a specialist interest in head and neck radiology, who was blinded to the pathology results. The Hounsfield units (HU) of the 100 parathyroid glands was measured using the region of interest setting on Sectra Workstation IDS7 Version 21.2 (Sectra AB, Linkoping, Sweden); this was then compared with the HU of an area of normal, adjacent thyroid tissue using the same region of interest setting. This was performed in the pre-contrast, arterial and venous phases to compare with each other and group the parathyroid glands into groups A, B, C and D, as shown in Figure 1.

In all these groups, the parathyroid glands demonstrated lower HU compared to adjacent thyroid tissue in the pre-contrast phase. The histology of all glands was checked, which demonstrated either adenoma or hyperplasia.

Type A: Parathyroid gland enhancement is higher than that of the thyroid gland in the arterial phase and lower in the delayed phase.

Type B: Parathyroid gland enhancement is higher than the thyroid gland in the arterial and delayed phases.

Type C: Parathyroid gland enhancement is lower than the thyroid gland in the arterial phase and lower in the delayed phase.

Type D: Parathyroid gland enhancement is lower than the thyroid gland in the arterial phase and higher in the delayed phase.

The mean HU and standard deviation of the diseased parathyroid tissue and normal adjacent thyroid tissue for pre-contrast, arterial and venous phases were calculated for each subgroup. The percentage change in HU was calculated between pre-contrast, arterial and venous phases in both the parathyroid gland and surrounding thyroid tissue in all groups, and the mean and median HU were calculated. Following the production of these generic descriptive statistics, data were analysed for evidence of statistical significance using the Mann-Whitney U test, as they followed a non-parametric distribution. The percentage differences in HU between the diseased parathyroid gland and surrounding thyroid tissue in both arterial and venous phases were compared with the pre-contrast values. The differences between the arterial and venous phases were also analysed similarly. IBM SPSS Statistics for Windows, Version 28 (IBM Corp. Armonk, NY, USA) was used.

## Results

Ninety-two patients were identified who underwent parathyroidectomy within the timeframe. Of these 92 patients, six were excluded as per the exclusion criteria; the remaining 86 patients met all criteria. Of the 86 patients, 100 parathyroid glands were removed and histological results were available. This included 67 patients with single gland disease, demonstrating adenomatous changes, and 19 patients with multi-glandular disease demonstrating hyperplastic changes. Following analysis of each patient’s 4DCT images, the patient was allocated either Group A, B, C or D, depending on their enhancement patterns (Figure 1).

**Figure 2:**
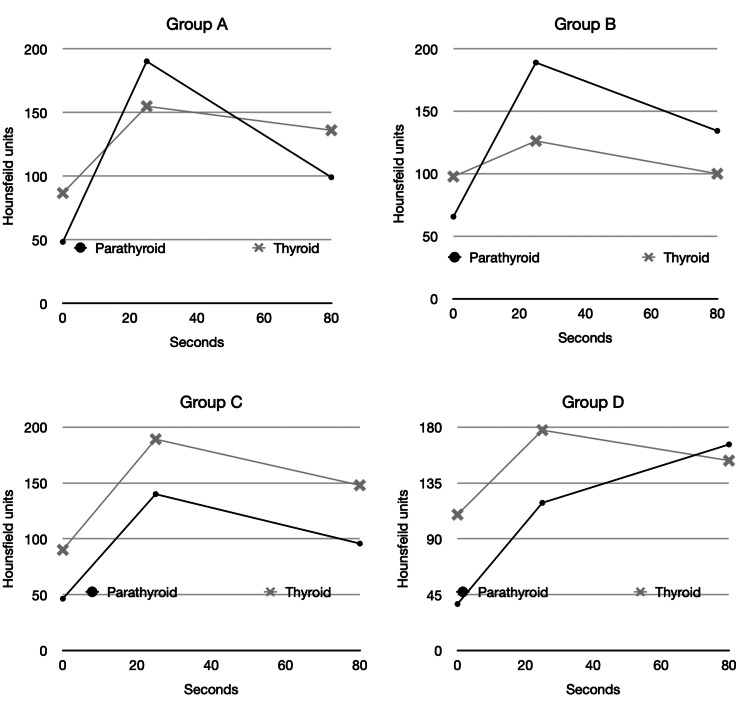
Graphical representation of the mean enhancement patterns of each group. The pre-contrast phase is at 0 seconds, the arterial phase at 25 seconds and the venous phase at 80 seconds.

The total number of patients in Group A was 35. The mean parathyroid value in the pre-contrast phase was 48.3 HU (SD = 25.8), the arterial phase 190.2 HU (SD = 54.7) and the delayed phase 99.1 HU (SD = 31.5) compared to the mean thyroid values of 86.7 HU (SD = 19.9) in the pre-contrast phase, 154.9 HU (SD = 36.5) in the arterial phase and 136.0 HU (SD 16.8) in the venous phase, as demonstrated in Figure 2. The total number of patients in Group B was 4. The mean parathyroid values were 65.6 HU (SD = 37.1) in the pre-contrast phase, 189.0 HU (SD = 25.2) in the arterial phase and 134.3 HU (SD = 30.1) in the venous phase. The mean thyroid values were 97.8 HU (SD = 39.0) in the pre-contrast phase, 126.3 HU (SD = 57.3) in the arterial phase and 100.0 HU (SD = 30.4) in the venous phase, as demonstrated in Figure 2. The total number of patients in Group C was 59. The mean parathyroid values were 46.4 HU (SD = 16.3) in the pre-contrast phase, 140 HU in the arterial phase (SD = 49.4) and 95.8 HU (SD = 34.6) in the venous phase. The mean thyroid values were 90.1 HU (SD = 24.3) in the pre-contrast phase, 189.1 HU (SD = 41.8) in the arterial phase and 147.9 HU (SD = 20.0) in the venous phase, as demonstrated in Figure 2. The total number of patients in Group D was 2. The mean parathyroid values were 37.5 HU (SD = 2.1) in the pre-contrast phase, 119 HU in the arterial phase (SD = 52.3) and 166 HU (SD = 1.4) in the venous phase. The mean thyroid values were 109.5 HU (SD = 24.7) in the pre-contrast phase, 177.5 HU (SD = 48.8) in the arterial phase and 153 HU (SD = 7.1) in the venous phase, as demonstrated in Figure 2.

The mean percentage change in HU values for the parathyroid and thyroid tissue was calculated between phases (Table 1), and the percentage change between parathyroid tissue and thyroid tissue was analysed for statistical significance. Significant statistical difference was found between the percentage change of HU between the diseased parathyroid gland and adjacent thyroid tissue between pre-contrast and arterial phases using *t*-test (*P *< 0.001), arterial and venous phases (*P *< 0.001) and pre-contrast and venous phases (*P *< 0.001), as demonstrated in Table 2.

**Table 1:** Mean percentage change between pre-contrast and arterial phases of parathyroid and thyroid glands in each group.

	A		B		C		D		All	
	Parathyroid	Thyroid	Parathyroid	Thyroid	Parathyroid	Thyroid	Parathyroid	Thyroid	Parathyroid	Thyroid
Mean percentage change	572	188	324	129	365	227	314	172	433	209

 

**Table 2:** Average percentage difference in HU between phases. HU, Hounsfield unit

	Average parathyroid percentage difference in HU (%)	Average thyroid percentage difference in HU (%)	*P*-value (Statistical significance)
Pre-contrast to arterial	330	192	<0.001
Pre-contrast to venous	111	63	<0.001
Arterial to venous	-39	-18	<0.001

## Discussion

In this study, all of the diseased parathyroid glands were found to be of lower attenuation when compared to the adjacent thyroid tissue during the pre-contrast phase. This is to be expected [7] and is due to the higher iodine content of the thyroid tissue, causing a higher density when compared with the lower-density parathyroid gland [10]. However, it is worth noting that in certain conditions, such as chronic thyroiditis, there may be a lower iodine content, meaning the density of the abnormal thyroid parenchyma is similar in attenuation to the parathyroid glands [11], which is a phenomenon that the reporting radiologist should be aware of. The arterial enhancement pattern of a parathyroid gland has been described by Doppman et al. [12] in the 1960s when they visualised arterial staining due to selective angiography of the inferior thyroid artery. This helped to form the basis of 4DCT scans for parathyroids, which was first described by Rodgers et al. [6]. The previously described characteristic enhancement pattern of the parathyroid gland is that it is higher in attenuation compared to the thyroid in the arterial phase, with a quicker washout causing a lower attenuation in the delayed phase - this is the Type A subgroup within our study. Although this well-described enhancement pattern is frequently quoted, several studies that have analysed this in detail have consistently found that other enhancement patterns also exist [7,9,13], matching our findings, although some group numbers were smaller. Our study further corroborates this finding, as only 35% of all diseased glands fall into the typical enhancement pattern (Type A). We found that the most common enhancement pattern was Type C with 59%, in which the parathyroid glands are lower in attenuation than the thyroid in both the arterial and delayed phases. It is worth noting, however, that the mean HU in the arterial phase in this subgroup is 140 HU, increasing from 46 HU in the pre-contrast phase, and that a more specific characteristic of the parathyroid glands was that there was a statistically significant increase in enhancement between the pre-contrast and arterial phases, with a mean percentage increase of 433% in the enhancement of parathyroid glands compared to a 209% increase in the enhancement of thyroid tissue. Similarly, there was a significant difference in the percentage change between pre-contrast and venous and arterial and venous phases between diseased parathyroid and thyroid tissue. This demonstrates that a percentage difference in HU may be more useful to identify parathyroid tissue, as well as to discriminate from lymph nodes, which enhances to a lesser extent [14]. These findings further highlight that relying solely on enhancement characteristics cannot reliably identify parathyroid glands. Instead, a thorough understanding of the normal location of the superior and inferior parathyroid glands is essential. Furthermore, due to the high occurrence of ectopic glands in patients with primary hyperparathyroidism [2], a robust knowledge of the embryological migration of the glands is paramount to ensure the identification of ectopic glands, which could easily be missed if not specifically looked for. As many radiologists can find reporting of 4DCT scans both challenging and time-consuming, particularly in the learning phase [7,15], we suggest that the reporting radiologist needs to have a systematic approach when reporting the images, which involves careful inspection of possible ectopic areas, and that this should be combined with the feedback of findings from the operating surgeon.

## Conclusions

The aim of this study - to identify and analyse the usefulness of different enhancement patterns on 4DCT - has been achieved. The study has demonstrated that not only is the classically described enhancement pattern of the parathyroid gland not always present, but it also is not even the most frequent. The relative enhancement characteristics between diseased parathyroid and adjacent thyroid tissue are variable and cannot be relied upon in isolation. Instead, a thorough understanding of anatomy, embryology and possible ectopic gland locations is essential. The study has also demonstrated a significant difference in the percentage change of HU in diseased parathyroid tissue and adjacent thyroid tissue between phases. This raises the question of whether there is a need for three phases and whether a two-phase scan, which would allow for a reduction in radiation for patients, maybe more beneficial if analysed using a percentage change in HU. Further studies could also look at possible explanations for altered enhancement patterns, as well as compare different imaging modalities. There is a learning curve to the reporting of 4DCT scans, and the reporting radiologist must have adequate knowledge of the embryology, anatomy and pathology of primary hyperparathyroidism, as lesions that look like parathyroids and are found in the expected anatomical or embryological positions may be the most important and consistent positive finding. Multi-disciplinary team meetings with radiologists, endocrinologists and surgeons to review images, along with the case information and operative findings, will provide feedback and improve the quality of care for patients.
